# The use of pulsed ultrasound with reduced power delivery to degrade the polysaccharide curdlan

**DOI:** 10.1016/j.ultsonch.2026.107747

**Published:** 2026-01-17

**Authors:** Eliza Malinowska, Michał Zmitrowicz, Grzegorz Łapienis, Jadwiga Turło

**Affiliations:** a Department of Drug Technology and Pharmaceutical Biotechnology, Medical University of Warsaw, 1 Banacha Str., 02-097 Warszawa, Poland; b National Centre for Nuclear Research Radioisotope Centre POLATOM, 7 Andrzeja Sołtana Str., 05-400 Otwock – Świerk, Poland; c Department of Functional Polymers and Polymeric Materials, Centre of Molecular and Macromolecular Studies, Polish Academy of Sciences, 112 Sienkiewicza Str., 90-363 Łódź, Poland

**Keywords:** Pulsed ultrasound, Degradation, Polysaccharides, Molecular weight, Curdlan

## Abstract

This study investigated the use of continuous ultrasound and low-power pulsed ultrasound on curdlan degradation by analyzing molecular weight changes during sonication. Although pulsed ultrasound only delivered one-sixth of the power of continuous ultrasound, it led to faster curdlan degradation. The most significant differences occurred within the first 25 min: Pulsed ultrasound accelerated the cleavage of polysaccharide chains, resulting in a degradation rate of approximately 60 % and a substantial reduction in the mass fraction of fragments with a molecular weight exceeding 400 kDa (from ca. 85 % to 3 %). Continuous ultrasound required 65 min to achieve a similar degree of degradation. The decrease in dispersity (from 1.13 to 1.06 within 65 min) indicated the non-random nature of the process, which occurred more rapidly during pulsed ultrasound. The degradation kinetics fit second-order and Ovenall/Harrington/Madras models, favoring pulsed ultrasound, which had a higher rate constant. Analysis of the chain scission mechanism showed a robust correlation between the midpoint scission model and the experimental data (*R*^2^ ∼ 0.96). According to the simulation analysis, larger curdlan particles are preferentially degraded, with pulsed ultrasound providing greater precision in cleavage localization. These findings suggest that employing pulsed ultrasound with a reduced power supply is an energy-efficient strategy to obtain more uniform polysaccharides with a moderately reduced molecular weight.

## Highlights

• Energy-efficient pulsed ultrasound outperforms continuous US in curdlan degradation.
• Pulsed US increases polysaccharide molecular weight reduction with less power.
• Ultrasonic curdlan degrades via near-midpoint scission, biased toward longer chains.
• Ultrasound-induced reduction in dispersity improves curdlan solution homogeneity.

## Graphical abstract

**Figure:**
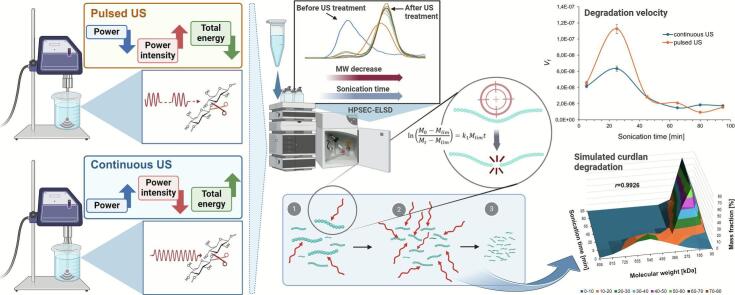

## 1 Introduction

In recent years, there has been a burgeoning research effort to explore the utility of ultrasound as a promising approach for the degradation of macromolecules, including bacterial and fungal polysaccharides [1], [2], [3]. These compounds are known for their multifaceted properties, particularly in immunomodulation, and their potential anticancer effects. In addition to specific structural attributes, critical factors that shape the physicochemical properties and biological activity of beta-glucans include their molecular weight and water solubility. Beta-glucans are particularly notable among polysaccharides due to their unique immunomodulatory properties. They directly interact with specific immune cell receptors, such as Toll-like receptor (TLR), dectin-1, and complement receptor 3 (CR3), triggering pathways that enhance innate and adaptive immunity. This ability to influence immune responses is a distinctive feature that sets beta-glucans apart from other polysaccharides and underpins their therapeutic potential, especially in oncology and infection control [4], [5]. There have been debates regarding the relationship between molecular weight and biological activity of beta-glucans [6], [7], [8]. Higher-molecular-weight beta-glucans are thought to have greater anticancer effects, yet they often show poor solubility and high viscosity, which limit their clinical utility. Conversely, some studies suggest equal or better results with highly soluble, lower-molecular-weight polysaccharides [9], with intermediate-molecular-weight beta-glucans showing promise [10]. Thus, in addition to their biological activity, beta-glucans stand out due to their distinct structural features and variable solubility, key factors that influence their clinical and nutritional potential.

An optimal approach to utilize beta-glucose for therapeutic purposes may involve breaking the polymer chain into water-soluble fragments of moderately reduced molecular weight while preserving chemical structure and bioactivity. Ultrasound stands out among various techniques for its effectiveness, environmental friendliness, and energy efficiency. Researchers have postulated that the propagation of ultrasonic waves within a solution triggers cavitation, followed by the rapid growth and subsequent collapse of microbubbles [11]. Ultrasonic degradation minimizes side reactions and monomer formation, without the need for a reaction initiator, unlike conventional chemical and enzymatic methods [12]. The fundamental principle of ultrasonic disruption involves delivering low-frequency, high-intensity energy to cleave chemical bonds, resulting in products with reduced molecular weight, improved water solubility, and enhanced susceptibility to subsequent chemical modifications, all of which contribute to enhance biological activity [13]. Consequently, ultrasound offers a promising approach to degrade complex polysaccharides into novel functional and bioactive units.

In this study, we examined the ultrasonic degradation of the bacterial polysaccharide curdlan, which is a notable food additive with emerging pharmacological potential [14]. Curdlan, first discovered in 1966, is a linear β-(1 → 3)-D-glucan produced by certain gram-negative bacteria that has traditionally been utilized in the food industry to form versatile gels; recently, however, the focus has pivoted toward its medicinal properties [15]. The pronounced insolubility of curdlan in water, alcohol, and most organic solvents, stemming from robust hydrogen bonding and a lack of hydrophilic side chains, constrains its direct applicability in therapeutic contexts [16]. The challenges associated with using curdlan to meet clinical requirements or its potential use as a functional food are significant obstacles that have driven the exploration of efficient strategies that can be used to produce depolymerized curdlan derivatives with broader potential applications [17], [18], [19], [20]. While enzymatic and chemical methods are frequently employed to obtain various oligosaccharides, they cannot be used effectively for curdlan due to its water insolubility. In a hydrolytic environment, linear curdlan chains self-assemble into a dense triple helix, resulting in cross-linking and resistance to hydrolysis [21]. On the other hand, excessive degradation can result in the creation of short fragments with significantly reduced biological activity [19]. Therefore, ultrasound, which enables the controlled production of polymer fragments with molecular weight limited only to some extent, appears to be the most rational choice. The application of pulsed ultrasound seems particularly promising. Researchers investigating the impact of ultrasound on macromolecule degradation have identified limitations associated with the continuous processing mode, including probe wear and energy inefficiency. Conversely, pulsed ultrasound can enhance cavitation effects by reducing the formation of bubble clusters that impede degradation, thereby potentially promoting chemical reactions [22], [23]. We aimed to utilize pulsed ultrasound to achieve similar or improved curdlan degradation relative to continuous treatment while delivering significantly less power to the solution. We compared and assessed the degradation of curdlan during continuous and low-power delivery of pulsed ultrasonic treatments by monitoring changes in molecular weight through high-performance size-exclusion chromatography coupled with an evaporative light scattering detector (HPSEC-ELSD). Additionally, we investigated the degradation rate, alterations in polydispersity, degradation kinetics, and the mechanism of chain cleavage. Our findings lay the groundwork for designing experiments to identify specific factors that enhance the efficiency of the degradation process for various linear polysaccharides of clinical and nutritional significance.

## 2 Materials and methods

### 2.1 Materials and chemicals

Curdlan derived from *Alcaligenes faecalis* was purchased from Immunocon Bioremedies Pvt. Ltd (Mumbai, India). A series of β-glucan molecular weight standards with established molecular weights was acquired from Megazyme (Bray, Ireland). Ammonium acetate, acetic acid, and ammonium hydroxide needed to prepare ammonium acetate buffer were purchased from Sigma-Aldrich (St. Louis, MO, USA). Aqueous solutions were prepared with ultra-pure water. All used reagents were of analytical grade.

### 2.2 Ultrasonic treatment

Curdlan (0.125 g) was suspended in 50 mL of 5 mM ammonium acetate buffer with a pH of 10.35. It was dissolved at 50 °C on a magnetic stirrer to produce a homogenous solution. The choice of ammonium acetate buffer as a solvent was based on its compatibility with the subsequent analysis of curdlan ultrasonic products using an ELSD. Additionally, this solvent ensured adequate solubility of curdlan. The sample solution was transferred to a glass vessel and subjected to ultrasonic treatment at a constant temperature of 50 ± 5 °C in an ice water bath; this treatment is sufficient for ultrasonic degradation, ensures the structural stability of curdlan, and minimizes undesirable thermal effects. Ultrasonic irradiation was performed using a UP 200St homogenizer (Hielscher Ultrasonics GmbH, Teltow, Germany) operating at 26 kHz. Each sample was prepared independently and subjected to ultrasonic treatment for a specific period of time, ranging from 5 to 95 min.

In the initial stage of the investigation, continuous ultrasonic irradiation was performed for 18 h; this process employed a homogenizer equipped with an ultrasound generator and a tip diameter of 14 mm. Samples were collected every 2 h throughout the entire treatment period. To reduce experimental error caused by uneven power transfer, the sample was positioned directly beneath the ultrasound source, and the sonotrode was immersed in the solution to half of its length (40 mm). The objective of this experiment was to assess the effectiveness of degradation, to determine the suitability of selected parameters and procedural conditions to ensure a stable process, and to identify the point at which additional changes in the polymer chain length cease to occur.

Next, we examined whether the use of pulsed ultrasound with lower power delivered to the solution instead of continuous ultrasound would cause similar or faster degradation of curdlan. For this purpose, both continuous and pulsed experiments were performed for 95 min, with the corresponding parameters outlined in Table 1. When ultrasound was emitted continuously (at a full-duty cycle), an ultrasound generator with a 14-mm-diameter tip was utilized. By immersing it to approximately half its length (40 mm) and setting the vibration amplitude to 100 %, 170 W of power was transmitted to the solution, with a power intensity of 104 W/cm^2^. In pulsed mode, ultrasound was generated with a 50 % duty cycle (meaning equal intervals between pulses, resulting in a 1:1 on/off ratio), a total cycle length of 1 s, and an amplitude set to 100 %. This treatment employed a 2-mm-diameter sonotrode submerged to half of its length (60 mm). This setup resulted in an average transmission of 28 W of power to the solution and a power intensity of 1019 W/cm^2^. A 50 % duty cycle was selected based on published data: It is suitable for the ultrasonic degradation of polysaccharides and other compounds [24], [25], [26], [27]. The sonotrode tip diameter and duty cycle allowed for a six-fold reduction in the power delivered to the solution compared with continuous ultrasonic treatment.

**Table 1:** Process parameters and experimental conditions for 95-min continuous and pulsed ultrasonic treatment.

Ultrasound mode	Sonotrode tip diameter [mm]	Average power [W]	Power intensity [W/cm^2^]	Total energy [kJ]	Duty cycle
Continuous	14	170	104	969	100 %
Pulsed	2	28	1019	160	50 %

### 2.3 Molecular weight determination and calculation of dispersity (Đ)

HPSEC-ELSD (equipment from Shimadzu, Kyoto, Japan) was employed to monitor the degradation process. Prior to analysis, all collected samples were passed through glass fiber syringe filters with a diameter of 25 mm and a pore size of 1 µm (Chromafil GF-100/25, Macherey-Nagel, Düren, Germany), tailored for viscous solutions and enabling the removal of solid particles originating from the sonotrode. Subsequently, 20 µL of the analyzed solutions were injected into the column. The analysis was conducted at 50 ℃ using two connected Ultrahydrogel linear columns (7.8 mm × 300 mm) from Waters (Milford, Massachusetts, USA), along with an Ultrahydrogel pre-column (6 mm × 40 mm). The mobile phase consisted of 5 mM ammonium acetate buffer at pH 10.35. Elution was performed at a flow rate of 0.5 mL/min, and detection was performed at 80 ℃. Calibration was performed using a series of β-glucans with known molecular weights, each at a concentration of 2.5 mg/mL. *Đ*, defined as the ratio of the weight-average molecular weight to the number-average molecular weight (*M_w_/M_n_*), was calculated using the SEC Analyser Software (Version 1.0.8285.40629).

### 2.4 Calculation of the degradation rate

The degradation rate, corresponding to changes in the molecular weight of curdlan during ultrasonic treatment, was determined using the following formula [28]:

(1) $$Degradation\;Rate=\frac{M_{0}-M_{t}}{M_{0}}\times 100\%$$

where $M_{0}$ and $M_{t}$ represent the initial molecular weight and the molecular weight at a certain treatment time, respectively.

### 2.5 Calculation of the velocity of curdlan degradation

The following derivative of the reciprocal molecular weight as a function of the ultrasonic treatment time was used to describe how fast curdlan degrades within ultrasonic irradiation:

(2) $$V_{f}\left(t_{i}\right)\approx \frac{f\left(t_{i+1}\right)-f\left(t_{i}\right)}{t_{i+1}-t_{i}}$$

where

(3) $$f\left(t\right)=\frac{1}{M_{w}\left(t\right)}$$

$f\left(t\right)$ is the reciprocal of the weight-average molecular weight as a function of degradation time, and $V_{f}\left(t_{i}\right)$ is the average rate of change of f at a specific period $t_{i}$ of ultrasonic treatment.

### 2.6 Degradation kinetics of curdlan under continuous and pulsed ultrasonication

First- and second-order kinetic models [28] were fitted to the experimental data from the ultrasonic treatment of curdlan. The degree of fit was analyzed based on the relationship between molecular weight and degradation time, as defined by the following general equation:

(4) $$\frac{d[M]}{\mathrm{dt}}=-{k[M]}^{n}$$

Ultrasonic degradation follows first-order kinetics when *n* = 1. Thus:

(5) $$\int_{M_{0}}^{M_{t}}\frac{1}{\left[M\right]}d\left[M\right]=-k_{1}\int_{0}^{t}dt$$

(6) $$\ln\frac{M_{t}}{M_{0}}=k_{1}t$$

To describe the degradation behavior of curdlan in accordance with second-order degradation kinetics (*n* = 2), the following equation was used:

(7) $$\int_{M_{0}}^{M_{t}}\frac{1}{{\left[M\right]}^{2}}d\left[M\right]=-k_{2}\int_{0}^{t}dt$$

The above equation was derived by Malhotra [29], taking its final form:

(8) $$\frac{1}{M_{t}}-\frac{1}{M_{0}}=k_{2}t$$

where *k*_1_ is the first-order kinetic rate constant, (min^−1^), *k*_2_ is the second-order kinetic rate constant (mol g^−1^ min^−1^), *t* is the treatment time (min), $M_{0}$ represents the initial molecular weight of curdlan, and $M_{t}$ is the molecular weight of curdlan at a specific treatment time.

Additionally, we fit the Ovenall/Harrington/Madras (OHM) model to the experimental data [30], [31], [32]. According to Akyüz et al. [33], this model effectively describes the ultrasonic degradation of polymers in solution, capturing the characteristic behavior of an initial rapid decline in molecular weight, followed by a slower rate of molecular weight reduction toward a limiting value (*M*_lim_) where polymer scission no longer occurs. The model is represented by:

(9) $$\ln\left(\frac{1}{M_{\lim}}-\frac{1}{M_{t}}\right)=\ln\left(\frac{1}{M_{\lim}}-\frac{1}{M_{0}}\right)-k_{\mathrm{OHM}}\left(\frac{M_{\lim}}{cm_{\mathrm{mon}}}\right)t$$

where $M_{0}$ and $M_{\lim}$ are the initial and limiting molecular weights, $M_{t}$ is the instantaneous molecular weight as a function of the sonication time *t*, *c* is the polymer concentration, $m_{\mathrm{mon}}$ is the monomer molecular weight and $k_{\mathrm{OHM}}$ is the rate constant.

### 2.7 Chain scission models for ultrasonic degradation of curdlan

The mid-chain and random scission models were employed to elucidate the mechanism of curdlan chain cleavage. They can be expressed as shown in Eqs. (10), (11), respectively [34], [35], [36], [37]:

(10) $$\ln\left(\frac{M_{0}-M_{\lim}}{M_{t}-M_{\lim}}\right)=k_{3}M_{\lim}t$$

(11) $$\frac{M_{\lim}}{M_{t}}+\ln\left(1-\frac{M_{\lim}}{M_{t}}\right)={-\frac{k_{4}}{c}t\left(\frac{M_{\lim}}{m}\right)}^{2}+\frac{M_{\lim}}{M_{0}}+\ln\left(1-\frac{M_{\lim}}{M_{0}}\right)$$

where $M_{0}$, $M_{t}$, and $M_{\lim}$ denote the initial molecular weight, the molecular weight at a given ultrasonic time *t*, and the limiting molecular weight, respectively; $k_{3}$ is the degradation rate constant for the midpoint scission model and *k*_4_ is the degradation rate constant for the random scission model; *c* is the initial concentration of polymer solution; and *m* is the molecular weight of the monomer.

### 2.8 Analysis of the mass fraction with different molecular weights

The ELSD response function was integrated to determine the percentage mass fractions of curdlan within distinct molecular weight ranges for each sample collected at successive time intervals of ultrasonic treatment, according to the following formula:

(12) $$\mathrm{Mass}\;\mathrm{Fraction}=\frac{\int_{{\mathrm{RT}}_{n_{1}}}^{{\mathrm{RT}}_{n_{2}}}f_{t}\left(r\right)dr}{\int_{{\mathrm{RT}}_{n_{0}}}^{{\mathrm{RT}}_{n_{\max}}}f_{t}\left(r\right)dr}\times 100\%$$

where $f_{t}\left(r\right)$ is the detector response as a function of retention time *r*; ${\mathrm{RT}}_{n_{0}}$ indicates the lowest retention time in the elution profile; ${\mathrm{RT}}_{n_{\max}}$ is the highest retention time; ${\mathrm{RT}}_{n_{1}}$ represents the retention time corresponding to the lower limit of the integration in a given section of the elution profile; and ${\mathrm{RT}}_{n_{2}}$ is the retention time corresponding to the upper limit of the integration in a given interval of the elution profile.

Numerical integration by the trapezoidal rule method was used to approximate the solution of the above equation:

(13) $$\int_{{\mathrm{RT}}_{n_{1}}}^{{\mathrm{RT}}_{n_{2}}}f_{t}\left(r\right)dr\approx \sum_{i=n_{1}}^{n_{2}-1}\frac{h}{2}\left[f_{t}\left({\mathrm{RT}}_{i+1}\right)+f_{t}\left({\mathrm{RT}}_{i}\right)\right]$$

(14) $$\int_{{\mathrm{RT}}_{n_{0}}}^{{\mathrm{RT}}_{n_{\max}}}f_{t}\left(r\right)dr\approx \sum_{i=n_{0}}^{n_{\max}-1}\frac{h}{2}\left[f_{t}\left({\mathrm{RT}}_{i+1}\right)+f_{t}\left({\mathrm{RT}}_{i}\right)\right]$$

where:

(15) $$h={\mathrm{RT}}_{i+1}-{\mathrm{RT}}_{i}$$

${\mathrm{RT}}_{i}$ represents the specific retention time points at which the detector produces discrete signal values.

The percentage rate of change in the mass fraction (Δ*MF*) during ultrasonication was calculated for each molecular weight range at a specific treatment time *t* as follows:

(16) $$\Delta MF=\frac{\left(X_{t}-X_{t-1}\right)}{X_{0}}\times 100\%$$

where $X_{t}$ represents the mass fraction of fragments within the defined molecular weight range in the sample collected at a specific treatment time, $X_{t-1}$ – in the sample gathered from the previous measurement, and $X_{0}$ – in the initial sample. Eq. (13) was used to calculate $X_{t}$, $X_{t-1}$, and $X_{0}$, with the integration limits specified as retention time points. These points were derived by translating the molecular weight ranges, determined from the calibration curve of the β-glucan standards, into corresponding retention times. The relationship between molecular weight ranges and retention times is detailed in Table S1.

### 2.9 Simulation of curdlan degradation

An iterative model was used to simulate the degradation of molecules through ultrasonic treatment (see the Supplementary Material). The model assumed a constant mass of particles degraded per unit of time, while the emergence and efficiency of cavitation bubbles were treated as stochastic processes, introducing a non-deterministic element to the simulation. The optimization process involved an examination of the influence of various parameters, including cavitation intensity, the preferential molecular mass range subject to degradation, the minimum molecular mass after cleavage, and the degree of chain cleavage asymmetry. The model also integrated phenomena such as the preferential cleavage of larger molecules and the intensified degradation of more abundant groups. The simulation results were validated against experimental data based on the Pearson correlation coefficient (*r*) and the sum of squared differences. Each simulation scenario was repeated 50,000 times with various parameter combinations.

The simulation incorporated preferential cleavage of larger particles, allowing precise definition of the molecular weight range subject to simultaneous degradation. The model also accounted for preferences toward larger particle groups and imposed constraints based on a minimum particle mass, effectively preventing the formation of particles smaller than a specified threshold. The molecular weight distribution during the cleavage process can occur symmetrically (halving) or with a certain degree of asymmetry, depending on the specified parameters.

The simulation procedure involved the following steps.

1. Examination of the elution profile: The entire elution profile was divided into a predefined number of molecular mass ranges. The response surface within each range was summed to determine the initial mass distribution.
2. Iterative mass calculation: The mass degraded during each iteration (iterative mass) was calculated as the quotient of the total particle mass, the duration of ultrasonic treatment, and the number of iterations per minute. This parameter could be adjusted using an intensity coefficient to enhance the accuracy of the simulated results.
3. Cleavage simulation: Weight functions were generated by using a random number generator for each molecular weight range undergoing degradation. The iterative mass was distributed among the ranges based on these weights, creating partial iterative masses. These masses were subtracted from the respective ranges, while the resulting fragments were added to smaller ranges as degradation products.
4. Iterations: The cleavage simulation was repeated for the duration of ultrasonic treatment, multiplied by the number of iterations per minute. Simulated molecular mass distributions were recorded at time points corresponding to the collection of physical samples.
5. Comparison of the results: Once the simulations were completed, the molecular mass distributions were compared with the experimental results to evaluate the accuracy of the model.

## 3 Results and discussion

### 3.1 Use of ultrasound for curdlan degradation

**Fig. 1:**
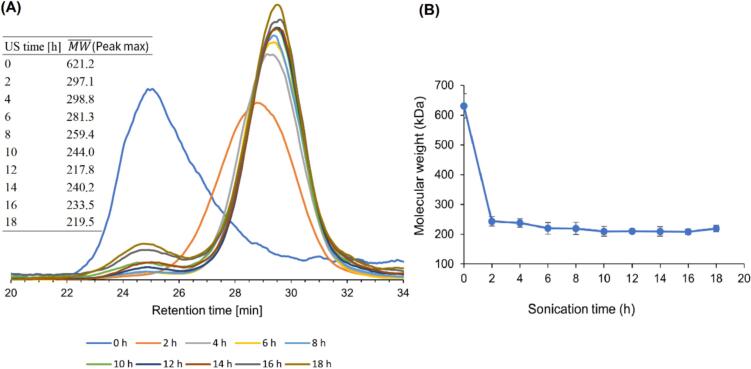
High-performance size-exclusion chromatography (HPSEC) profiles and molecular weight evolution of curdlan during extended (18-h) continuous ultrasonication. (A) HPSEC profiles before and after different ultrasonic treatment durations. The embedded table displays molecular weights corresponding to major peaks ($\overset{¯}{\mathrm{MW}}$ refers to molecular weight at the peak maximum). (B) The change in molecular weight during ultrasonic degradation. US, ultrasound.

To assess the use of ultrasound on the depolymerization of curdlan under predefined conditions and to identify the point at which degradation ceased, we examined molecular weight alteration during an 18-h ultrasonic treatment. The elution profile of the initial curdlan sample revealed a signal at a retention time of 24.66 min (Fig. 1A), corresponding to a molecular weight of approximately 620 kDa (the average calculated from the peak maximum). When we employed ultrasound, the peak shifted toward lower molecular weights.

As depicted in Fig. 1B, curdlan was degraded rapidly within the first 2 h, followed by a near cessation of degradation. During this period, the molecular weight decreased approximately 2-fold—from the initial value of about 620 kDa to less than 300 kDa—accompanied by a decrease in the *M_w_*/*M_n_* ratio (Fig. S1). After approximately 6 h, the molecular weight of the degraded curdlan reached a final value of about 210 kDa (the average of measurements obtained from 2 to 18 h of ultrasonic treatment). This value corresponds to the limiting molecular weight (*M_lim_*), below which polymers do not undergo scission. Irrespective of how long sonication continues past this stage, the weight-average molecular weight must not decrease below *M_lim_* [34].

Our observations are consistent with a well-known phenomenon in which low-frequency ultrasound (20–100 kHz) reduces the molecular weight of polysaccharides through mechanical degradation that occurs near collapsing cavitation bubbles. The rapid decrease in molecular weight at the beginning of sonication indicates that long polymer chains fragment quickly due to the shear forces generated by cavitation collapse. This finding aligns with previous studies on the ultrasonic treatment of high-molecular-weight polysaccharides [37], [38], [39]. Compounds with a higher molecular weight are more prone to fragmentation because they can absorb more kinetic energy from the ultrasonic waves, facilitating the breaking of the polymer chains. In contrast, smaller molecules with shorter relaxation times exhibit greater resistance to sonication-induced stresses [40].

### 3.2 Influence of the ultrasound generation mode on molecular weight, the degradation rate, and Đ

**Fig. 2:**
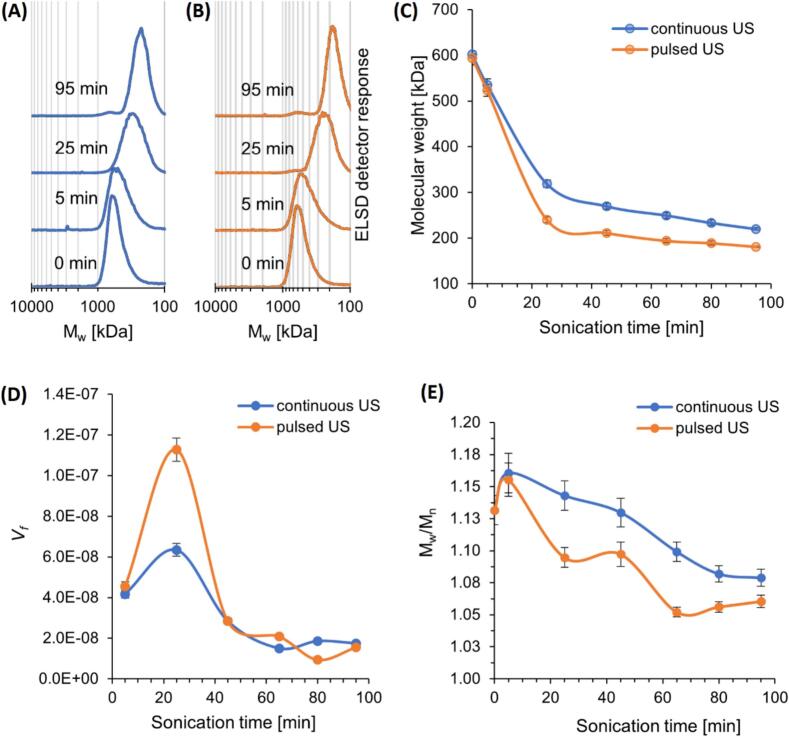
Characterization of curdlan during ultrasonication. High-performance size-exclusion chromatography (HPSEC) elution profiles after selected sonication times under (A) continuous and (B) low-power pulsed ultrasonic treatment. (C) Change in molecular weight under continuous and low-power pulsed ultrasonic treatment. (D) Degradation velocity as a function of sonication time. (E) Changes in dispersity of degraded curdlan. US, ultrasound.

We investigated whether intermittent ultrasonic wave transmission (50 % duty cycle) could lead to similar or better curdlan degradation while reducing energy consumption compared with continuous ultrasonic wave transmission. Specifically, we investigated changes in the molecular weight of curdlan following ultrasonic treatment. Both pulsed and continuous ultrasonic treatment lasted up to 95 min, with individually prepared samples treated at different intervals. The key difference between the two treatments was the type of sonotrode employed: The pulsed ultrasound sonotrode had a smaller surface area, resulting in a higher ultrasonic energy density and a sixfold reduction in the power delivered to the solution compared with the sonotrode used in the continuous mode. This experimental setup allowed us to compare the effectiveness of pulsed ultrasound, with lower power input, against continuous ultrasound, which, despite having a larger sonotrode and higher power delivery, had a lower energy density. The chromatographic separation of curdlan treated with continuous and low-power pulsed ultrasound revealed a progressive shift in signal toward lower molecular weights (Fig. 2A and 2B).

The molecular weight of curdlan decreased faster during pulsed ultrasonic treatment than during constant ultrasonic treatment. After 25 min, the molecular weight reached ca. 250 kDa for pulsed ultrasonic treatment, while the molecular weight was 350 kDa for continuous ultrasonic treatment (Fig. 2C). As shown in Table 2, the degradation degree of low-power pulsed ultrasonic treatment after 25 min (60 %) closely resembled the degradation degree of continuous ultrasonic treatment after 65 min (59 %). The stabilization phase commenced after 65 min of sonication. By 95 min, the degradation degree was approximately 7 % higher for pulsed ultrasonic treatment compared with continuous ultrasonic treatment.

**Table 2:** Alterations in the degradation degree of curdlan after continuous and low-power delivery pulsed ultrasonic treatment.

Time [min]	Degradation degree [%]	
	Continuous ultrasound	Pulsed ultrasound
5	11.16 ± 2.04^a^	11.89 ± 2.14^a^
25	47.08 ± 1.27^b^	59.60 ± 1.46^d^
45	55.20 ± 1.19^c^	64.45 ± 1.46^ef^
65	58.55 ± 1.17^cd^	67.33 ± 1.37^fg^
80	61.24 ± 1.16^de^	68.20 ± 1.34^g^
95	63.47 ± 1.28^e^	69.54 ± 1.16^g^

The values are presented as the mean ± standard deviation (n = 3). Different lowercase letters indicate a significant difference (p < 0.05) within both rows and columns, reflecting cross-comparisons.

Analysis of the velocity of curdlan degradation provided additional evidence that pulsed ultrasonic treatment was more effective than continuous ultrasonic treatment. Fig. 2D illustrates differences in the impact of continuous and low-power pulsed ultrasonic treatment on the degradation velocity of curdlan. During the first 25 min of treatment with pulsed ultrasound, polysaccharide chain cleavage began to accelerate rapidly, reaching twice the speed achieved with continuous ultrasound. Continuous ultrasonic treatment never reached the degradation velocity of pulsed ultrasonic treatment. After 45 min, the molecular weight reduction rate for continuous ultrasound reached the same level as that achieved with pulsed ultrasound; it remained comparable until the end of the process. Consequently, we concluded that curdlan degradation through pulsed ultrasonic treatment is more effective than continuous ultrasonic treatment, despite the inactive periods in ultrasound delivery and reduced power supplied to the solution. Based on the results, we speculate that even a sixfold decrease in the power delivered to the solution during the pulsed generation of ultrasound is sufficient to produce a superior curdlan-degrading effect. However, further research is required to determine the ultrasonic power threshold at which degradation will continue to occur to the same extent.

The enhanced efficiency of pulsed ultrasound highlights the critical influence of the physical parameters involved in the process, the phenomena arising from the intermittent operating cycle, and the evolving dynamics of cavitation. The use of a sonotrode with a smaller diameter reduced the power delivered to the solution but increased the energy flux. Thus, this approach had a more substantial cavitation effect, so degradation was more intense and long curdlan chains were broken down more effectively. These outcomes meet our primary objective of minimizing energy consumption while ensuring high degradation efficiency. The increased intensity of pulsed ultrasound, leading to a higher number of cavitation events in aqueous polysaccharide solutions (such as increased cavitation energy, a lower cavitation threshold, and an increased number of cavitation bubbles), has been discussed by other authors [23], [27], [41], [42], [43]. Although continuous ultrasonic treatment delivers more energy to the solution, the lower intensity per unit area of the sonotrode means the curdlan chain degrade more slowly, which may explain the delayed achievement of similar degradation results compared with pulsed ultrasonic treatment.

Some theories also suggest that more complex mechanisms are involved in the greater efficiency of pulsed ultrasound compared with continuous ultrasound, although they do not provide a definitive explanation for this phenomenon. According to Henglein et al. [44], the augmentation of cavitation activity during pulsed ultrasound operation stems from the behavior of the remaining cavitation nuclei during the inactive portion of the pulse. Cavitation bubbles exist in various sizes, but only those with specific dimensions can generate the extreme conditions (high temperature and pressure) required to break down the chemical bonds of a polymer. Larger bubbles tend to move toward the nodes of sound pressure distribution, ultimately failing to enhance degradation. In contrast, smaller bubbles can readily combine to form clusters, which create a shielding effect that inhibits degradation. Employing pulsed ultrasound reduces the accumulation of bubbles during inactive periods, thus promoting chemical reactions.

HPSEC-ELSD analysis revealed that the initial curdlan exhibited non-uniform characteristics as a polysaccharide, displaying a moderately polydisperse nature, as indicated by the calculated *Đ* of 1.13. Fig. 2E illustrates the alterations in *Đ* as sonication progressed. There was a short-term increase in *Đ* during the first 5 min of the process, irrespective of the ultrasonic operating mode, and the broadening of the curdlan peak in the elution profile (Fig. 2A and 2B) may indicate the beginning of degradation, wherein some particles have undergone depolymerization while others have remained intact. As the process continued, the curdlan peak began to narrow, suggesting the formation of a set of homogeneous particles with either the same or very similar molar masses. We observed this trend throughout the remaining period under continuous ultrasonic treatment, eventually reaching a low *Đ* of 1.08. *Đ* reached a comparable value (1.06) with low-power pulsed ultrasound treatment. Nevertheless, the reduction occurred more rapidly—within 65 min—compared with continuous ultrasonic treatment. This trend indicates an accelerated degradation process, resulting in products with comparable chain lengths and a narrow molecular weight distribution. A similar variation pattern in *Đ*, characterized by an initial increase, a rapid peak attainment and a subsequent decline, was also identified in research concerning the ultrasonic degradation of pullulan [45].

Emsley and Heywood [46] postulated that changes in molecular weight distribution depend on the chain scission mechanism. While random scission does not alter the *M*_w_/*M*_n_ ratio, backbone cleavage near the center of the polymer chain decreases this ratio. A shift in *Đ* from a value typical for moderately polydisperse macromolecules (1.13) to one closer to monodisperse polymers (1.08) suggests a non-random degradation mechanism with cleavage near the chain center and aligns with findings from a previous study [39]. The more pronounced *M*_w_/*M*_n_ ratio decline during pulsed ultrasonic treatment results from a faster transition of long polymer chains from the higher molecular weight range to the lower molecular weight range. This result suggests that pulsed ultrasonic treatment may be more effective for producing more uniform polysaccharide solutions with a narrower molecular weight distribution.

### 3.3 Degradation kinetics of curdlan under ultrasonic treatment

Next, we undertook a more thorough evaluation of curdlan degradation efficacy during ultrasonic treatment by utilizing well-documented and validated mathematical models. Additionally, we performed a more detailed comparison of the effects of continuous and low-power pulsed ultrasonic treatment. For this purpose, we characterized the degradation behavior using first-order, second-order, and OHM kinetic models [28], [30], [31], [32].

Table 3 presents the determination coefficients (*R*^2^) for ln(*M*_t_/*M*_0_) (first-order reaction kinetics), 1*/M*_t_ (second-order reaction kinetics), and ln(1/*M*_lim_-1/*M*_t_) (OHM model) in relation to the ultrasonic treatment time *t* for continuous and pulsed operation. The larger coefficients indicate that the second-order kinetic model provides a better description of the curdlan degradation process than the first-order kinetic model, and the changes in molecular weight align well with the Malhotra model expressed in Eq. (8). According to this model, in the initial stages of ultrasonic treatment, the molecular weight decreases rapidly as longer chains are readily fragmented, mainly due to their heightened susceptibility to mechanical stress. As the process progresses, the degradation rate declines due to the increasing presence of shorter chains, which are more resistant to further breakdown. The good fit of this kinetic model aligns with the degradation kinetics of other polysaccharides, such as *Flammulina velutipes* polysaccharide, schizophyllan, and sea cucumber fucoidan [27], [43], [47]. However, the model lacks a limiting molecular weight.

**Table 3:** Degradation rate constants (*k*) and determination coefficients (*R*^2^) from first-order, second-order, and Ovenall/Harrington/Madras (OHM) kinetic models for curdlan solution subjected to continuous and low-power pulsed ultrasonic treatment.

Ultrasound mode	First order		Second order			OHM	
	*k*_1_	*R*^2^		*k*_2_	*R*^2^	*k_OHM_*	*R^2^*
Continuous	4.55 **×** 10^−3^	0.885		3.05 **×** 10^−8^	0.925	2.60 **×** 10^−2^	0.970
Pulsed	5.73 **×** 10^−3^	0.822		4.37 **×** 10^−8^	0.894	3.69 **×** 10^−2^	0.962

The OHM model proved to be the most suitable for describing the degradation kinetics of curdlan. This model accounts for the rapid initial drop in molecular weight, followed by a gradual decrease to a limiting molecular weight, below which no further degradation occurs [33]. Researchers have shown that this model effectively describes the scission kinetics of some synthetic linear polymers, such as poly(vinylpyrrolidone) (PVP) and polyacrylamide [31], [48]. The degradation rate constant *k_OHM_*, determined by linear regression fitting of the ln(1/*M*_lim_-1/*M*_t_) data against sonication time *t*, was higher for pulsed ultrasonic treatment, indicating greater efficiency in breaking down the curdlan chain. These findings could result from the pronounced cavitation process and an increased likelihood of polysaccharide degradation during pulsed ultrasonic treatment.

### 3.4 Chain scission models for ultrasonic degradation of curdlan

**Fig. 3:**
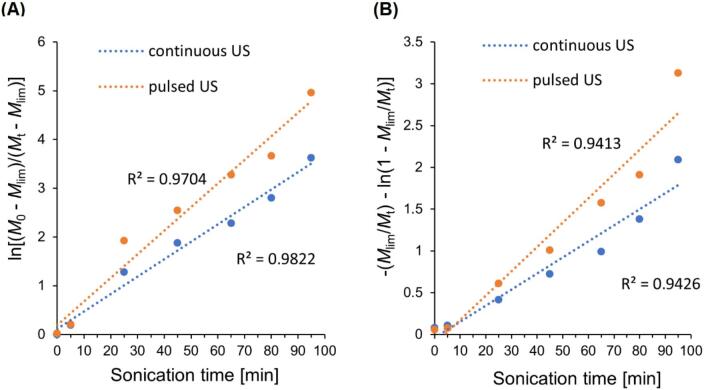
Comparison between (A) the midpoint scission model and (B) the random scission model for curdlan subjected to continuous and low-power delivery pulsed ultrasonic treatment. R^2^, determination coefficient; US, ultrasound.

We wanted to gain a better understanding of the mechanism behind curdlan chain cleavage and the location of this cleavage, so we fit the experimental data with models in which the polymer ultimately approaches the limiting molecular weight. Among these simplified models, the model of mid-chain scission (*P(x) → 2P(x/*2*)*) posits that chain breaks occur centrally within the polymer backbone, as represented by Eq. (10) [35], [36]. The model developed by Schmid [37] postulates that chain degradation occurs randomly (*P(x) → P(x – x') + P(x')*), and each of the links forming the chain have an equal chance of breaking, as described by Eq. (11). Fig. 3 presents the relationship between ultrasonic time *t* and the respective models of curdlan degradation, specifically $\ln[\left(M_{0}-M_{\lim}\right)/\left(M_{t}-M_{\lim}\right)]$ versus *t* for the midpoint scission model (Fig. 3A) and $-\left(M_{\lim}/M_{t}\right)-\ln\left(1-M_{\lim}/M_{t}\right)$ versus *t* for the random scission model (Fig. 3B).

The linear correlation of the midpoint scission model significantly surpasses that of the random scission model. The high *R*^2^ obtained from linear regression analysis confirms the effectiveness of the midpoint scission model in accurately predicting the experimental data. The adjusted *R*^2^ of 0.982 and 0.970 for continuous and pulsed ultrasonic treatment, respectively, indicates a strong fit between the model and the observed results.

The fact that the near-midpoint scission model provides a good explanation for curdlan degradation may stem from the specific hydrodynamic forces generated during cavitation. As ultrasound propagates through the solution, cavitation bubbles form and rapidly collapse, creating high-gradient shear fields. These fields cause polymer segments near the collapsing bubbles to move faster than those farther from the cavity. This difference in movement induces internal stresses within the polymer chain, leading to scission. Consequently, polymers undergo non-random scission, with the most probable point of fracture occurring near the center of the chain. The observed curdlan degradation behavior aligns with findings reported by other researchers, who hypothesized that ultrasonic degradation of various linear polymers follows the near-midpoint scission model [45], [49], [50], in contrast to highly branched polysaccharides, which degrade more chaotically [1], [50], [51].

### 3.5 Changes in the distribution of curdlan fragments over different molecular weight ranges

**Fig. 4:**
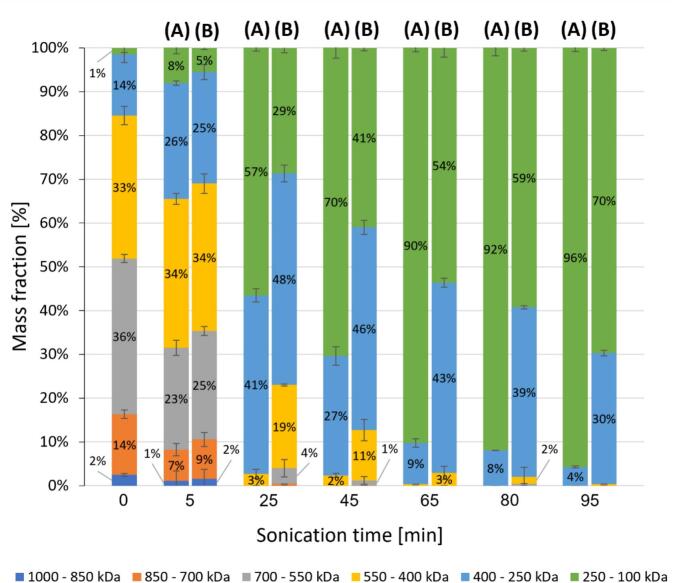
Changes in the mass fractions of curdlan fragments within different molecular weight ranges during sonication with (A) low-power delivery pulsed ultrasound and (B) continuous treatment. The mass fractions have been rounded to whole numbers.

The assessment of mass fractions across different molecular weight ranges has enabled more precise tracking of changes in the proportions of degraded curdlan fragments during ultrasonic treatment, providing valuable insights into the influence of ultrasonic treatment on the molecular weight reduction of curdlan through chain scission. Moreover, it has supplied additional information regarding the pace of these changes and the efficiency of low-power delivery pulsed ultrasound. Fig. 4A and 4B depict the percentage fluctuations of curdlan fragments within each molecular weight category under continuous and pulsed ultrasonic treatment, respectively. After 25 min of ultrasonic treatment, there was an evident distinction in the efficacy of both ultrasound generation methods. In the initial curdlan sample, the majority of the fractions consisted of large- and medium-sized molecules with a molecular weight exceeding 400 kDa (approximately 85 % in total). The application of pulsed ultrasonic treatment resulted in an almost twofold increase in the number of fractions with a molecular weight of 100–250 kDa compared with continuous ultrasonic treatment. Conversely, the overall mass fraction of chains with a molecular weight above 400 kDa decreased significantly to a mere 3 %, compared with nearly 20 % with the use of continuous ultrasonic treatment. In other words, pulsed ultrasonic treatment of curdlan for 25 min yielded the same effect as continuous ultrasonic treatment of curdlan for 65 min. We observed this effect throughout the entire operating ultrasonication time: After 5 min, there was a slightly higher proportion of smaller fractions after pulsed ultrasonic treatment compared with continuous ultrasonic treatment, which indicates the greater efficiency of pulsed ultrasound, despite the delivery of less power to the solution.

Next, we calculated Δ*MF* for each molecular weight range by using Eq. (16). Δ*MF* indicates whether there has been a rise or a fall in different molecular weight ranges compared with the preceding value. A negative value implies a decrease, whereas a positive value suggests an increase. As shown in Table 4, the most significant increase in Δ*MF* occurred for the molecules with a molar mass of 100–250 kDa after 25 min of ultrasonic treatment, regardless of the type of ultrasound. However, Δ*MF* was twice as high for pulsed ultrasonic treatment compared with continuous ultrasonic treatment. For pulsed ultrasonic treatment, there was a significant decrease in Δ*MF* during the initial sonication stages (within the first 25 min) that was considerably diminished in the subsequent phases. This pattern was in sharp contrast to continuous ultrasonic treatment, where the effects persisted for at least 65 min. These findings strongly support the notion of the superior effectiveness of low-power pulsed ultrasonic treatment.

**Table 4:** Rate of change in the mass fraction (Δ*MF*) of curdlan fragments with different molecular weights under continuous and low-power pulsed ultrasonic treatment.

The values are presented as the mean ± standard deviation (n = 3). Different lowercase letters in the same column indicate a significant difference (p < 0.05). The negative Δ*MF* values are highlighted in gray.

### 3.6 Simulated curdlan degradation

Although our Δ*MF* analysis substantiated the higher efficiency of low-power pulsed ultrasonic treatment, significantly reducing the time required to achieve results comparable to continuous ultrasonic treatment, it also provided ambiguous insights into the depolymerization sequence of curdlan chains. Specifically, it remained uncertain whether the longer chains undergo preferential depolymerization before the shorter ones or whether depolymerization co-occurs across chains of varying lengths. The changes in the proportions of curdlan fragmentation products (Fig. 4, S2, and S3), occurring during ultrasonic treatment across all considered molecular weight ranges, along with the variations in Δ*MF* (Table 4), suggest that depolymerization is not confined solely to the highest molecular weight fragments. Instead, shorter chains may begin the fragmentation process even before all longer chains have completely degraded. Within just 5 min of initiation, both continuous and pulsed ultrasonic treatment appear to trigger the degradation of curdlan chains across all molecular weight ranges, excluding the shortest fragments (i.e., 100–250 kDa). The degradation of chains above 550 kDa is unequivocally indicated by a negative Δ*MF*, while one could infer this for fractions in the 250–550 kDa range based on the minimal increase compared with fractions below 250 kDa. It is plausible that intermediate-molecular-weight fractions experience a simultaneous increase and decrease, leading to a delicate balance near zero and consequently explaining the increase in the quantity of the smallest fractions. After 25 min of ultrasonic treatment, we observed ongoing changes in the levels of all fractions simultaneously. Beginning at 45 min, the longest curdlan fragments began to diminish in favor of shorter ones with a molar mass below 250 kDa.

Most studies on the effects of ultrasound on high-molecular-weight polysaccharides indicate a preferential depolymerization of longer chains [27], [52]. This preference can be attributed to the greater vulnerability of these longer chains to ultrasound, as they are more susceptible to mechanical stress and have an increased likelihood of cleavage [53].

Our simulation revealed the presence of preferential fragmentation of larger particles, although the phenomenon was broader than initially anticipated, encompassing molecules with masses within a range of approximately 600 kDa. This finding suggests that degradation does not target a specific molecular mass; rather, it exhibits a wider effective range. The results indicate that more abundant groups are not subject to intensified degradation. In the pulsed mode, fragmentation occurs closer to the center of symmetry of the molecule, with a deviation of 6 % and a minimum molecular mass of 218 kDa. In the continuous mode, the deviation reaches 23 %, with a minimum molecular mass of 252 kDa, indicating that this mode is less precise and efficient. The higher efficiency of pulsed ultrasonic treatment may stem from the greater local energy intensity of cavitation.

**Fig. 5:**
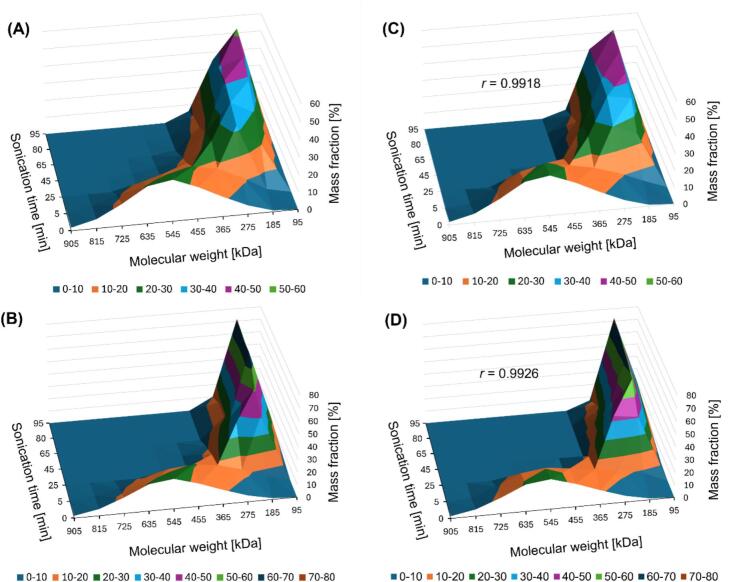
Three-dimensional plot of changes in the mass fractions of curdlan fragments with different molecular weights during ultrasonication: (A) based on experimental data from continuous processing; (B) based on experimental data from pulsed processing; (C) theoretical plot with optimal simulation parameters for continuous processing; and (D) theoretical plot with optimal simulation parameters for pulsed processing. *r*, Pearson correlation coefficient.

The final optimization of parameters achieved high concordance between the simulation results and the empirical data, confirmed by an *r* exceeding 0.99 for both types of ultrasonic treatment (Fig. 5). Incorporating the entire elution profile, rather than focusing solely on the maximum molecular masses within a given peak, provided greater analytical precision. This approach represents a significant advantage over traditional mathematical models. The iterative simulation method simplifies implementation and offers flexibility in modeling various phenomena and behaviors across different experimental scenarios. The application of this model could contribute to a better understanding of molecular degradation processes in diverse chemical systems and their optimization for industrial purposes.

## 4 Conclusions

Applying pulsed ultrasound enhances curdlan degradation more effectively than continuous ultrasound despite delivering six times less power to the solution. The degradation effects are particularly pronounced during the initial phase of pulsed ultrasonic treatment and stabilize more rapidly than with continuous ultrasonic treatment. The higher ultrasonic intensity and phenomena resulting from the duty cycle enhance cavitation, facilitating the breakdown of long curdlan chains into intermediate-length fragments while minimizing energy consumption.

The reduction in *Đ* and adherence to the mid-chain scission model suggests that during ultrasonic treatment, the most likely mechanism of curdlan degradation is a non-random process that occurs near the center of the polymer chain. The faster decrease in *Đ* and the higher degradation rate constant *k* underscore the effectiveness of pulsed ultrasound in achieving a more uniform curdlan solution with narrower molecular weight distributions.

The OHM model satisfactorily describes the curdlan degradation process under ultrasonic treatment. It indicates a nonlinear polymer depolymerization toward the limiting molecular weight, below which further degradation does not occur. Curdlan degradation preferentially targets larger particles within a broad molecular weight range. The cleavage point of curdlan chains depends on the type of ultrasonic treatment. Pulsed ultrasound, which generates intense energy pulses, leads to less asymmetric cleavage.

In summary, low-power pulsed ultrasonic wave transmission is more efficient and cost-effective for curdlan depolymerization. This technique holds promise for practical applications extending beyond curdlan, enabling the generation of other linear polysaccharide derivatives with intermediate molecular weights. Future research should prioritize identifying the critical factors in pulsed ultrasonic treatment responsible for curdlan degradation and extending this method to other linear beta-glucans. This research should encompass structural and physicochemical analyses, coupled with an assessment of the biological activity of polysaccharide derivatives with reduced molecular weight obtained under carefully optimized conditions.

## CRediT authorship contribution statement

**Eliza Malinowska:** Writing – original draft, Methodology, Investigation, Data curation, Conceptualization. **Michał Zmitrowicz:** Visualization, Software, Formal analysis. **Grzegorz Łapienis:** Writing – review & editing, Visualization. **Jadwiga Turło:** Supervision, Project administration.

## Funding

This research did not receive any specific grant from funding agencies in the public, commercial, or not-for-profit sectors.

## Declaration of competing interest

The authors declare that they have no known competing financial interests or personal relationships that could have appeared to influence the work reported in this paper.
