# Supplementary material for: The use of pulsed ultrasound with reduced power delivery to degrade the polysaccharide curdlan
Source: Ultrason Sonochem. 2026 Jan 17;125:107747. doi: 10.1016/j.ultsonch.2026.107747 (PMC12861279; doi:10.1016/j.ultsonch.2026.107747)
Supplement: Supplementary Data 1 [file mmc1.docx]

**SUPPLEMENTARY MATERIAL**

**The use of pulsed ultrasound with reduced power delivery to degrade the polysaccharide curdlan**

Eliza Malinowska^*a^ (e-mail: eliza.malinowska@wum.edu.pl), Michał Zmitrowicz^b^ (e-mail: michal.zmitrowicz@polatom.pl), Grzegorz Łapienis^c^ (e-mail: grzegorz.lapienis@cbmm.lodz.pl), Jadwiga Turło^a^ (e-mail: jadwiga.turlo@wum.edu.pl)

^a^Department of Drug Technology and Pharmaceutical Biotechnology, Medical University of Warsaw, 1 Banacha Str., 02-097 Warszawa, Poland

^b^National Centre for Nuclear Research Radioisotope Centre POLATOM, 7 Andrzeja Sołtana Str., 05-400 Otwock – Świerk, Poland

^c^ Department of Functional Polymers and Polymeric Materials, Centre of Molecular and Macromolecular Studies, Polish Academy of Sciences, 112 Sienkiewicza Str., 90-363 Łódź, Poland

*Corresponding author: Eliza Malinowska, 1 Banacha Str., 02-097 Warszawa, Poland, e-mail: eliza.malinowska@wum.edu.pl, phone +48225720647

**Fig. S1.** Plot of M_w_/M_n_ of curdlan against sonication time during 18 h of continuous ultrasonic treatment.

**Fig. S2.** Changes in the percentage contribution of individual curdlan fragments during 95 min of low-power pulsed ultrasonic treatment. Curves with different colors represent fragments detected at different retention times in SEC analysis.

**Fig. S3.** Changes in the percentage contribution of individual curdlan fragments during 95 min of continuous ultrasonic treatment. Curves with different colors represent fragments detected at different retention times in SEC analysis.

**Table S1.** Retention times and their corresponding molecular weight values of curdlan fragments in specified sections of the SEC elution profile. Frames of different colors encompass various molecular weight ranges.

| MW range | I | | III | | V | |  |
| --- | --- | --- | --- | --- | --- | --- | --- |
|  |  | II | | IV | | VI | |
| Retention time [min] | 22,7 | 23,4 | 24,2 | 25,3 | 26,7 | 28,7 | 32,7 |
| Molecular weight [kDa] | 1000 | 850 | 700 | 550 | 400 | 250 | 100 |

**A fragment of the code for the iterative model to simulate the degradation of curdlan molecules through sonolysis:**

for (size_t i = 0; i < Gdata->fractionData->sonolysisTime.size(); i++) //[time]

{

double sum = 0;

for (size_t j = 0; j < Gdata->fractionData->elutionTime.size(); j++) //[fraction]

{

sum += Gdata->fractionData->data[i][j];

}

for (size_t j = 0; j < Gdata->fractionData->elutionTime.size(); j++) //[fraction]

{

if (j == 0) {

if (i == 0) {

totalFraction = sum * (double)numericUpDownSimuInten->Value;

}

}

inputSum.push_back(Gdata->fractionData->data[i][j]);

if (i == 0) {

fractions.push_back(Gdata->fractionData->data[i][j]);

randomness.push_back(0);

outputSum.push_back(Gdata->fractionData->data[i][j]);

}

}

}

double time = Gdata->fractionData->sonolysisTime[0];

double increment = 1 / (double)numericUpDownSimuCountIM->Value;

int id = 1;

// Initialize pseudo-random number generator

std::random_device rd;

std::mt19937_64 generator(rd());

// Define random range

std::uniform_int_distribution<int> distribution(1, 1000);

do

{

double randomSum = 0;

double simSum = 0;

int radius = (int)numericUpDownSimuPrefRadius->Value;

for (size_t j = 0; j < Gdata->fractionData->elutionTime.size() - 1; j++)

{

randomness[j] = distribution(generator);

randomSum += randomness[j];

simSum += fractions[j];

}

for (size_t j = 0; j < Gdata->fractionData->elutionTime.size() - 1; j++)

{

if (checkBoxSimuPrefer->Checked)

randomness[j] = (randomness[j] / randomSum);

else

randomness[j] = (randomness[j] / randomSum) * (fractions[j] / simSum);

}

for (size_t j = 0; j < Gdata->fractionData->elutionTime.size() - 1; j++)

{

if (fractions[j] <= 0) continue;

if (checkBoxSimuPrefer->Checked) radius--;

if (radius < 0) continue;

double removal = randomness[j] * totalFraction;

fractions[j] -= removal;

if (fractions[j] < 0) {

removal += fractions[j];

fractions[j] = 0;

}

bool assigned = false;

for (size_t n = 0; n < Gdata->fractionData->elutionTime.size(); n++)

{

if (Gdata->fractionData->range[j].Fod / 2 > Gdata->fractionData->range[n].Fdo) {

fractions[n] += removal;

assigned = true;

break;

}

}

if (!assigned && checkBoxSimuCutoff->Checked)

{

fractions[Gdata->fractionData->elutionTime.size() - 1] += removal;

}

assigned = false;

for (size_t n = 0; n < Gdata->fractionData->elutionTime.size(); n++)

{

if (Gdata->fractionData->range[j].Fdo / 2 > Gdata->fractionData->range[n].Fdo) {

fractions[n] += removal;

assigned = true;

break;

}

}

if (!assigned && checkBoxSimuCutoff->Checked)

{

fractions[Gdata->fractionData->elutionTime.size() - 1] += removal;

}

}

if (id < Gdata->fractionData->sonolysisTime.size() && time >= Gdata->fractionData->sonolysisTime[id] - increment)

{

double sum = 0;

for (size_t j = 0; j < Gdata->fractionData->elutionTime.size(); j++) //[fraction]

{

sum += fractions[j];

outputSum.push_back(fractions[j]);

}

for (size_t j = 0; j < Gdata->fractionData->elutionTime.size(); j++)

{

}

id++;

}

time += increment;

} while (time <= Gdata->fractionData->sonolysisTime.back());

double correlation = boost::math::tools::correlation_coefficient(inputSum, outputSum);
